# The Innovative Medicines Initiative neurodegeneration portfolio: From individual projects to collaborative networks

**DOI:** 10.3389/fneur.2022.994301

**Published:** 2022-11-02

**Authors:** Diana O'Rourke, Nina Coll-Padrós, Angela Bradshaw, Lewis Killin, Laurent Pradier, Jean Georges, Dalia M. Dawoud, Lennert Steukers, Carlos Diaz

**Affiliations:** ^1^National Institute for Health and Care Excellence, Manchester, United Kingdom; ^2^SYNAPSE Research Management Partners, Barcelona, Spain; ^3^Alzheimer Europe, Luxembourg, Luxembourg; ^4^Sanofi, Paris, France; ^5^National Institute for Health and Care Excellence, London, United Kingdom; ^6^Faculty of Pharmacy, Cairo University, Cairo, Egypt; ^7^Janssen Pharmaceutica NV, Beerse, Belgium

**Keywords:** neurodegeneration, collaboration, public-private partnership, research policy, IMI

## Abstract

The IMI public-private partnership between the European Commission and the European Federation of Pharmaceutical Industries and Associations (EFPIA) was launched in 2008 with an initial budget of €2 billion. Aiming to accelerate the development of innovative medicines for areas of unmet clinical need, the IMI has committed over €380 million to projects on neurodegenerative disorders (NDD), catalyzing public-private collaborations at scale and at all stages of the R&D pipeline. Because of this vast investment, research on neurodegenerative diseases has made enormous strides in recent decades. The challenge for the future however remains to utilize this newly found knowledge and generated assets to develop better tools and novel therapeutic strategies. Here, we report the results of an integrated programme analysis of the IMI NDD portfolio, performed by the Neuronet Coordination and Support Action. Neuronet was launched by the IMI in 2019 to boost synergies and collaboration between projects in the IMI NDD portfolio, to increase the impact and visibility of research, and to facilitate interactions with related initiatives worldwide. Our analysis assessed the characteristics, structure and assets of the project portfolio and identifies lessons from projects spanning preclinical research to applied clinical studies and beyond. Evaluation of project parameters and network analyses of project partners revealed a complex web of 236 partnering organizations, with EFPIA partners often acting as connecting nodes across projects, and with a great diversity of academic institutions. Organizations in the UK, Germany, France and the Netherlands were highly represented in the portfolio, which has a strong focus on clinical research in Alzheimer's and Parkinson's disease in particular. Based on surveys and unstructured interviews with NDD research leaders, we identified actions to enhance collaboration between project partners, by improving the structure and definition of in-kind contributions; reducing administrative burdens; and enhancing the exploitation of outcomes from research investments by EU taxpayers and EFPIA. These recommendations could help increase the efficiency and impact of future public-private partnerships on neurodegeneration.

## Introduction

The Innovative Medicines Initiative (IMI) is a public-private partnership between the European Union (EU) and the European pharmaceutical industry, represented by the European Federation of Pharmaceutical Industries and Associations (EFPIA). It was approved in December 2007 with a €2 billion budget, and subsequently renewed for the period 2014–2020 as IMI2, with a budget of up to €3.276 billion. The overarching mission of the IMI is “to improve health by accelerating the development of, and patient access to, innovative medicines, particularly where there is an unmet medical or social need.” IMI aims to achieve its mission through the facilitation of engagement and collaboration between key stakeholders involved in healthcare research, such as universities, industry, small- and medium-sized enterprises (SMEs), patient organizations, and medicines regulators (1).

To address the key challenges facing the European healthcare systems, the pharmaceutical industry and regulatory agencies, IMI2 has focused its research across 12 priority disease areas, including neurodegenerative diseases (NDDs) for which there is a lack of available therapeutic interventions, despite high levels of research expenditure (2). In its Strategic Research Agenda (SRA), IMI2 identified several key areas of focus, including increased mechanistic understanding of NDDs, improved frameworks for risk factor screening, and innovative trials for disease prevention and treatment.

Guided by its Strategic Governance Group (SGG) on neurodegeneration, IMI2 has funded a diverse portfolio of projects in these focus areas. Projects such as PD-MitoQUANT, IMPRiND, PHAGO and ADAPTED address the molecular underpinnings of Parkinson's and Alzheimer's disease, while RADAR-CNS, RADAR-AD, IDEA-FAST and Mobilise-D are focused on digital assessment and endpoints across several NDDs. AMYPAD, a sister project to EPAD, is evaluating the role and relevance of amyloid imaging biomarkers across the dementia risk spectrum, while PD-MIND is trialing a repurposed, nicotinic agonist drug for Parkinson's disease. Together with EPAD, EMIF and AETIONOMY (IMI1 neurodegeneration projects that ended recently), these projects represent a breadth of research that covers the entire translational science spectrum, from preclinical research in cells and animal models to applied, clinical research involving human participants.

While initially planned as complementary concepts during the development of call topics and texts by the Strategic Governance Groups of the IMI, the diverse range of projects funded by the IMI bears the risk of excessive segmentation and lack of interaction between projects, limiting the impact of individual results and projects. To mitigate this risk, in March 2019 the NEURONET initiative was established to provide a platform for promoting collaboration, communication and synergies across the range of IMI funded neurodegenerative disease projects. This three-year Coordination and Support Action, which receives €1,199,125 in funding through IMI2, aims to maximize the impact of the portfolio as a whole by enhancing the visibility of project outputs and assets and creating active connections between projects and with other global research initiatives.

As both NEURONET and the IMI2 programme come to an end, it is a valuable opportunity to reflect on the lessons learnt and successes of the IMI NDD programme to inform future public-private partnership research programmes, including IMI's successor the Innovative Health Initiative (IHI, https://www.ihi.europa.eu/). In this article the NEURONET Consortium presents the results of an integrated analysis of the characteristics and structure of the project portfolio, provides an overview of assets generated by the projects, and reports on the lessons learned from past collaboration attempts between projects.

## Methods

### Identification of IMI NDD projects in scope

Firstly, we identified the IMI NDD projects that would be included within the scope of the analysis. All “neurodegenerative disease” or “Alzheimer's disease” related IMI projects were considered for inclusion in the portfolio. However, it was agreed to focus on active or upcoming projects, or projects that had finished within a year of the start date of NEURONET, in order to focus on the creation of synergies between present and future projects. Eighteen projects were identified and included in this analysis. We undertook an integrated programme analysis of the scope and impact of the 18 projects that are currently part of the IMI NDD portfolio, based on publicly available information, project documentation, interviews and survey results. These include 15 IMI2 projects and three IMI1 projects that have recently ended (Table 1).

**Table 1:** IMI neurodegenerative disease projects and calls.

**Project**	**IMI call**	**Call topic description**	**Duration**
EMIF	IMI1 CALL 4	A European medical information framework (EMIF) of patient-level data to support a wide range of medical research	January 2013–June 2018
AETIONOMY	IMI1 CALL 8	Developing an etiology-based taxonomy of human disease: Approaches to develop a new classification for neurodegenerative disorders with a focus on Alzheimer's disease and Parkinson's disease	January 2014–December 2018
EPAD	IMI1 CALL 11	European platform to facilitate proof of concept for prevention in Alzheimer's disease (EPOC-AD)	January 2015–October 2020
PRISM	IMI2 CALL 3	Linking clinical neuropsychiatry and quantitative neurobiology	April 2016–September 2019
RADAR-CNS	IMI2 CALL 3	Remote assessment of disease and relapse – CNS (part of the RADAR programme)	April 2016–March 2022
PHAGO	IMI2 CALL 5	Inflammation and ad: modulating microglia function – focussing on TREM2 and CD33	November 2016–April 2022
AMYPAD	IMI2 CALL 5	Understanding the role of amyloid imaging biomarkers in the current and future diagnosis and management of patients across the spectrum of cognitive impairment (from pre-dementia to dementia)	October 2016–September 2022
MOPEAD	IMI2 CALL 5	Evolving models of patient engagement and access for earlier identification of Alzheimer's disease: phased expansion study	October 2016–December 2019
ADAPTED	IMI2 CALL 5	From ApoE biology to validated Alzheimer's disease targets	October 2016–September 2020
ROADMAP	IMI2 CALL 6	Real world outcomes across the ad spectrum (ROADS) to better care (part of the BD4BO programme)	November 2016–October 2018
IMPRIND	IMI2 CALL 7	Identification of druggable targets modulating misfolded proteins in Alzheimer's and Parkinson's diseases	March 2017–February 2022
EQIPD	IMI2 CALL 9	Data quality in preclinical research and development	October 2017–September 2021
RADAR-AD	IMI2 CALL 12	Development and validation of technology enabled, quantitative and sensitive measures of functional decline in people with early stage Alzheimer's disease (RADAR-AD)	January 2019–June 2022
IM2PACT	IMI2 CALL 12	Discovery and characterization of blood-brain barrier targets and transport mechanisms for brain delivery of therapeutics to treat neurodegenerative & metabolic diseases	January 2019–December 2023
MOBILISE-D	IMI2 CALL 13	Linking digital assessment of mobility to clinical endpoints to support regulatory acceptance and clinical practice	April 2019–March 2024
PD-MITOQUANT	IMI2 CALL 13	Mitochondrial dysfunction in neurodegeneration	February 2019–July 2022
PD-MIND	IMI2 CALL 13	Pilot programme on a clinical compound bank for repurposing: neurodegenerative diseases	May 2019–April 2022
NEURONET	IMI2 CALL 13	Support and coordination action for the projects in the neurodegeneration area of the Innovative Medicines Initiative	March 2019–August 2022
IDEA-FAST	IMI2 CALL 15	Digital endpoints in neurodegenerative and immune-mediated diseases	November 2019–April 2025

### Data collection

We first identified a set of project parameters (Supplementary Figure 1) to be collected from the projects, including their scope and relative specialization, funding, participants, and outputs and assets. To collect this information, we developed a structured data collection form that was piloted with a subgroup of NEURONET partners for clarification and consistency. Firstly, we extracted information from publicly available sources, including the IMI website (https://www.imi.europa.eu), the CORDIS portal (https://cordis.europa.eu) and project websites. Where information was not available from these sources, we gathered information from the projects' Descriptions of Action/Work (DoA/DoW), newsletters, deliverables and other project reports.

Unstructured interviews were conducted with the leaders of 11 projects in the portfolio^1^, to gain a more in-depth understanding of those projects and to understand the lessons learned from past cross-project collaborations. Following these interviews, a survey was sent to 8 IMI NDD projects (ADAPTED, AETIONOMY, AMYPAD, EMIF, EPAD, IMPRiND, PHAGO and the related EBiSC project) to map and evaluate 16 attempted cross-project collaborations. The projects were asked for information on:

1. the topic of the collaboration;
2. whether the results of the collaboration were satisfactory or not;
3. whether legal support was required to materialize the collaboration, and
4. whether there were any specific obstacles hindering the collaboration.

Finally, we undertook a content analysis of project presentations from the NEURONET Annual Event at the 2019 Alzheimer Europe Conference. All project information was then combined into a single document (“project dossier”) which was validated by key representatives from each project to ensure completeness and accuracy. Understanding of project aims and status, as well as lessons learned and opportunities for collaboration, has been also continuously enhanced thanks to regular portfolio meetings gathering project leaders (under a “Scientific Coordination Board”) and other project participants (under “Working Groups” devoted to four specific, common issues found on most projects: data sharing, ethics and privacy, HTA/regulatory interactions and sustainability, as well as a “Communications Experts's Group composed of project managers and communications officers).

### IMI NDD portfolio analysis

To understand the structure and characteristics of the IMI NDD project portfolio, we conducted an integrated analysis of key metrics collected from the 18 IMI NDD projects, collected using the methods detailed in the previous section. Information was collated on every unique partner organization in the portfolio, including their organization type [Academic, EFPIA, Regulatory Agency, HTA body, patient/carer organization, SMEs, research funder, contract management organization (CMO), other] and the projects that they participate in. These data were used in network analyses (see below) and were analyzed in Microsoft Excel for the portfolio analysis.

The key information gathered about the IMI NDD portfolio has been summarized and collated through the publicly available NEURONET Knowledge Base (https://kb.imi-neuronet.org). The Knowledge Base was designed as an entry portal to the IMI NDD portfolio, providing a comprehensive overview of the breadth of IMI-funded NDD research, including detailed information about each project such as their objectives, deliverables and publications. The Knowledge Base also hosts interactive versions of the network analysis diagrams.

### Network analyses

A network analysis was conducted to characterize the connections between partner organizations and projects across the portfolio. Network analyses were performed using *R 4.1.0* (3) and the *igraph* [v1.2.6; (4)] package t. Specifically, a project-by-participant incidence matrix was used to create bipartite network graphs that represent the extent to which projects or participants are connected to others (i.e. “degree”), the structural relationship between those projects or participants (i.e. “betweenness”) and the strength of those connections (i.e., “weight”). In the case of the latter, this represents the number of projects that two participants collaborate on, or, conversely, the number of participants who all work on the same two projects.

Three network analyses were performed: (1) a network to show how partner organizations are connected to each other; (2) a partner network, with and without EFPIA partners; and (3) a project network. In the partner network, nodes represent each unique partner organization in the portfolio and the lines between them represent the number of projects that connect individual organizations. Nodes in the project network represent individual IMI NDD projects, and the connections between them the number of partner organizations that participate in both projects.

To assess the relative importance of a partner organization within the network, two measures of centrality were calculated: the ‘degree centrality’ and “betweenness centrality” (5). The betweenness centrality represents the number of times a node is present in the shortest path between two nodes in the network. This provides an indication of the key organizations in the network in terms of their ability to facilitate dissemination and exchange of knowledge through their connections to different organizations. The degree centrality is the number of links that one organization has to all other organizations in the network, indicating the relative importance of an organization within that network.

### Qualitative analyses

The results from the survey and transcripts of interviews with project leaders, as well as all other information captured through meetings, were analyzed qualitatively, focussing on the key challenges and opportunities for improvement for project collaborations.

## Results

### Summary metrics of the IMI NDD portfolio

**Figure 1:**
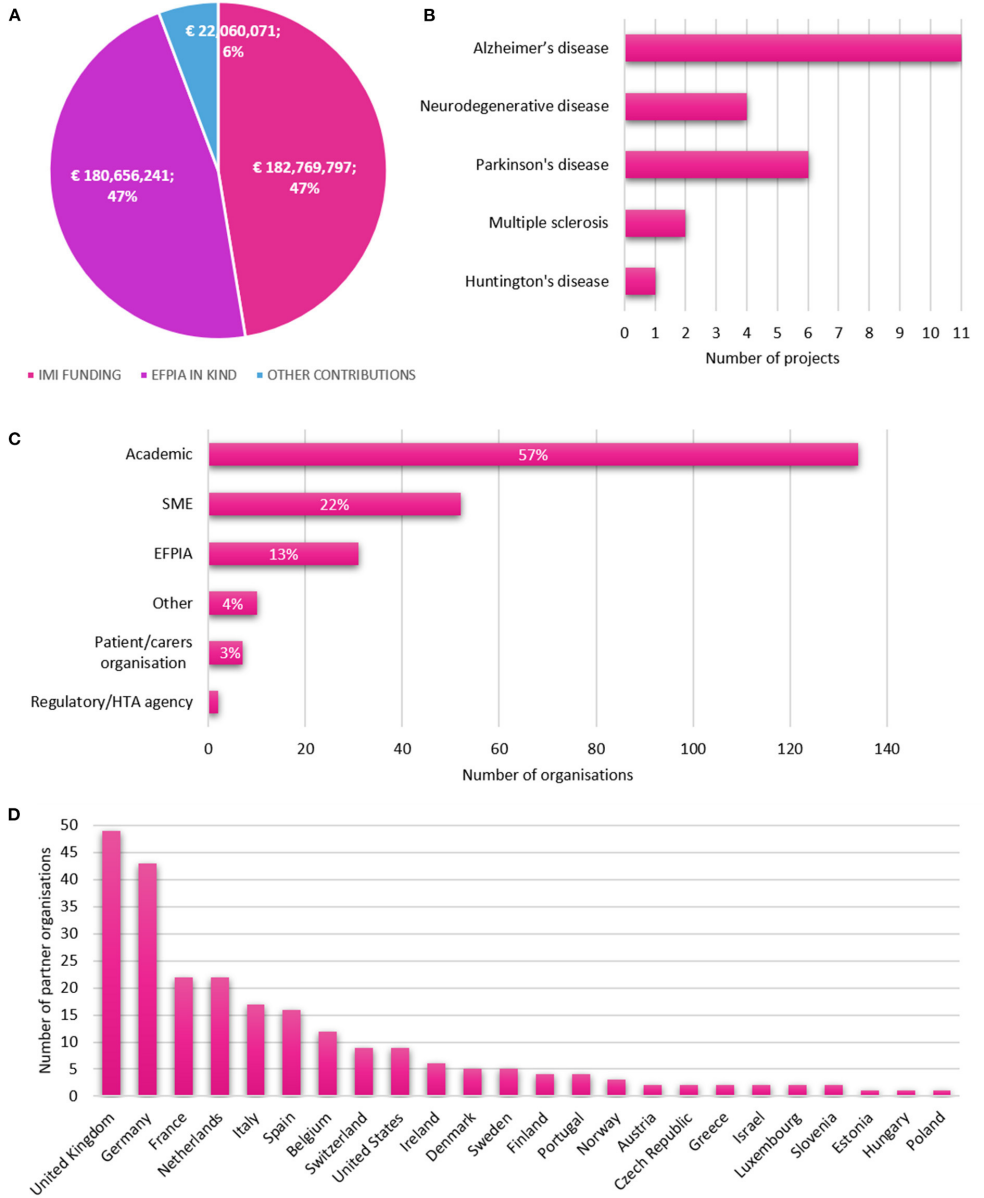
IMI neurodegenerative disease portfolio characteristics. A set of project parameters was collected for 18 projects in the IMI Neurodegenerative disease (NDD) portfolio from sources including EU databases, project websites and descriptions of work. **(A)** Total funding contributions for the IMI NDD portfolio, by source and amount. **(B)** Disease areas targeted by IMI NDD projects; number of projects. **(C)** Types of partner organisations in IMI NDD projects; number of organisations, by category. **(D)** Countries of partner organisations; by country.

The IMI NDD portfolio represents a total investment of €385.5 million, with the majority of funding coming from the EU and EFPIA (Figure 1A). There is a mean funding per project of €21,415,889.94. The 18 projects in the portfolio target a range of NDDs, however, the predominant focus is on Alzheimer's disease, with a secondary focus on Parkinson's disease (Figure 1B). Most of the projects in the portfolio are dedicated to the study of 1 specific NDD (*N* = 12). However, six projects (AETIONOMY, EQiPD, IDEA-FAST, IM2PACT, IMPRiND, Mobilise-D) cover several NDDs or are not focused on a particular NDD and have more general objectives (Tables 1, 2).

**Table 2:** IMI neurodegenerative disease project parameters.

**Project**	**Duration** **(months)**	**Partner** **organizations** **(*N*)**	**Total cost**	**Disease area**	**Website**	**Logo**
ADAPTED	48 months	13	€ 6,796,740	Alzheimer's disease	https://www.imi-adapted.eu	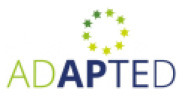
AETIONOMY	60 months	16	€ 17,812,216	Alzheimer's Parkinson's Neurodegenerative diseases	https://www.aetionomy.eu	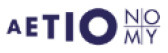
AMYPAD	54 months	15	€ 27,329,288	Alzheimer's disease	https://amypad.eu	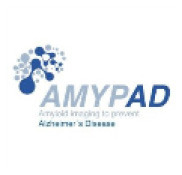
EMIF	54 months	60	€ 55,784,311	Alzheimer's disease	http://www.emif.eu	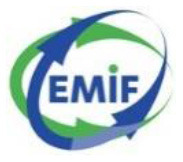
EPAD	57 months	39	€ 59,903,036	Alzheimer's disease	http://ep-ad.org/	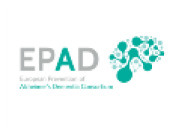
EQIPD	48 months	30	€ 9,360,692	Neurodegenerative diseases	https://quality-preclinical-data.eu	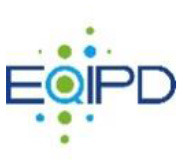
IDEA-FAST	66 months	51	€ 40,922,059	Huntington's disease Parkinson's disease	https://ideafast.eu	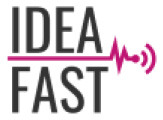
IM2PACT	60 months	27	€ 17,410,136	Neurodegenerative diseases	http://im2pact.org	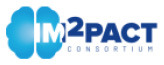
IMPRIND	60 months	18	€ 11,363,398	Alzheimer's disease Neurodegenerative diseases Parkinson's disease	https://www.imprind.org	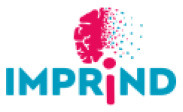
Mobilise-D	60 months	36	€ 49,361,564	Multiple sclerosis Parkinson's disease	https://www.mobilise-d.eu	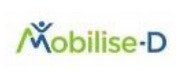
MOPEAD	39 months	15	€ 4,581,968	Alzheimer's disease	https://www.mopead.eu	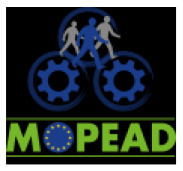
PD-MIND	36 months	10	€ 2,131,609	Parkinson's disease	https://www.pd-mind.org	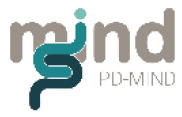
PD-mitoQUANT	42 months	14	€ 6,882,315	Parkinson's disease	https://www.pdmitoquant.eu/	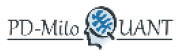
PHAGO	66 months	20	€ 18,088,176	Alzheimer's disease	https://www.phago.eu	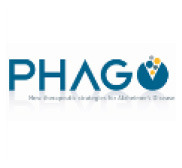
PRISM	42 months	23	€ 16,195,875	Alzheimer's disease	https://prism-project.eu	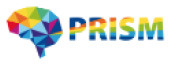
RADAR-AD	54 months	16	€ 7,640,145	Alzheimer's disease	http://www.radar-ad.org	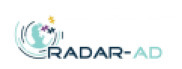
RADAR-CNS	66 months	25	€ 25,712,110	Multiple sclerosis	https://www.radar-cns.org	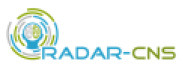
ROADMAP	24 months	26	€ 8,210,381	Alzheimer's disease	https://roadmap-alzheimer.org	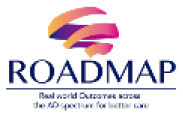

There are 236 unique partner organizations participating in the 18 projects in the IMI NDD portfolio. The majority of these organizations are academic institutions (*N* = 134), with a further 52 SME organizations and 31 EFPIA partners (Figure 1C). The majority of organizations (63%, *N* = 149) participate in a single project, including 41 SMEs, representing 79% of all SMEs in the portfolio. There is an average of 25 partners (range 10–60) per project.

Partner organizations are based across 24 different countries. Organizations from the UK (*N* = 48), Germany (*N* = 42), France (*N* = 22) and the Netherlands (*N* = 22) are most frequently represented (Figure 1D).

### Research focus and assets

From projects identifying new drug targets in Alzheimer's and Parkinson's disease, to the development of frameworks for access and assessment of real-world evidence, the IMI NDD portfolio covers a breadth of research and disease stages. Analysis of the 18 projects in the portfolio identified four projects that primarily focus on the identification and validation of novel targets through preclinical, mechanistic or *in vivo* research, including EQIPD, IM2PACT, IMPRIND and PD-Mitoquant. While IM2PACT, IMPRIND and PD-Mitoquant are characterizing specific disease mechanisms (blood-brain barrier dysfunction, protein aggregation and mitochondrial dysfunction, respectively), EQIPD has broader relevance across disease areas, establishing guidelines to strengthen the robustness, rigor and validity of research data. We identified three projects (ADAPTED, PHAGO, PRISM) involving translational research, spanning both preclinical and clinical stages of the drug development pipeline. For example, ADAPTED was focused on understanding the contribution of the apolipoprotein E (APOE) genetic risk factor to Alzheimer's disease, developing human cell models with disrupted APOE expression and investigating samples and data from patients with Alzheimer's disease.

We observed that the majority of IMI NDD projects were primarily focused on clinical research. Within these projects, PD-MIND is trialing a novel drug for the treatment of Parkinson's disease with mild cognitive impairment (MCI), while EMIF and EPAD have focused on developing large-scale cohort and electronic health record (EHR) studies on people at different stages of Alzheimer's development. Several projects are developing or testing news ways to detect and prognose NDDs, such as AMYPAD (amyloid imaging for Alzheimer's) and RADAR-AD, RADAR-CNS, IDEA-FAST and Mobilise-D (digital and/or gait biomarkers and endpoints). We observed that data assessment, access and sharing were a common focus across many clinical IMI NDD projects, with AETIONOMY organizing mechanistic knowledge on NDD, EMIF and EPAD developing methods for hosting and studying clinical study data, and ROADMAP creating a catalog and platform for real-world data access.

Since 2013, the 18 projects included in our analysis have developed a large number of assets, defined as tangible, accessible and re-useable project outputs that bring real value to the NDD research field. These assets are captured and depicted in the NEURONET Asset Map, a feature of the Knowledge Base that was developed following engagement with partners of the 18 IMI NDD projects. The Asset Map categorizes assets based on drug development pipeline stage (e.g., preclinical, clinical, real-world evidence) and asset type (e.g., datasets, cohorts, disease models, platforms and tools). Analyzing the 82 assets of the Asset Map, we observed that projects have developed a wide range of outputs, paralleling the breadth of the IMI NDD portfolio. As expected, given the clinical focus of IMI-funded NDD research, many of these assets are targeted at this stage of the drug development pipeline, including research cohorts (e.g., RADAR-CNS cohort of multiple sclerosis patients, EPAD longitudinal cohort study), patient samples and data (e.g., neuroimaging datasets from the AMYPAD studies, ADAPTED biosamples from people with different APOE genotypes) and tools for patient engagement, subject enrolment and clinical data analysis. The most well-populated area on the asset map, covering all stages of the drug development pipeline, was the category of “Tools, templates and guidelines,” with eight IMI NDD projects generating assets that could help progress preclinical research, clinical research recruitment, and stakeholder engagement with regulators and HTA. Perhaps reflecting the challenges of NDD drug development, with few new treatments for NDD reaching the market in the last 20 years, we only identified three accessible, re-useable assets on real-world evidence or targeted at regulators.

### Partner network analysis

**Figure 2:**
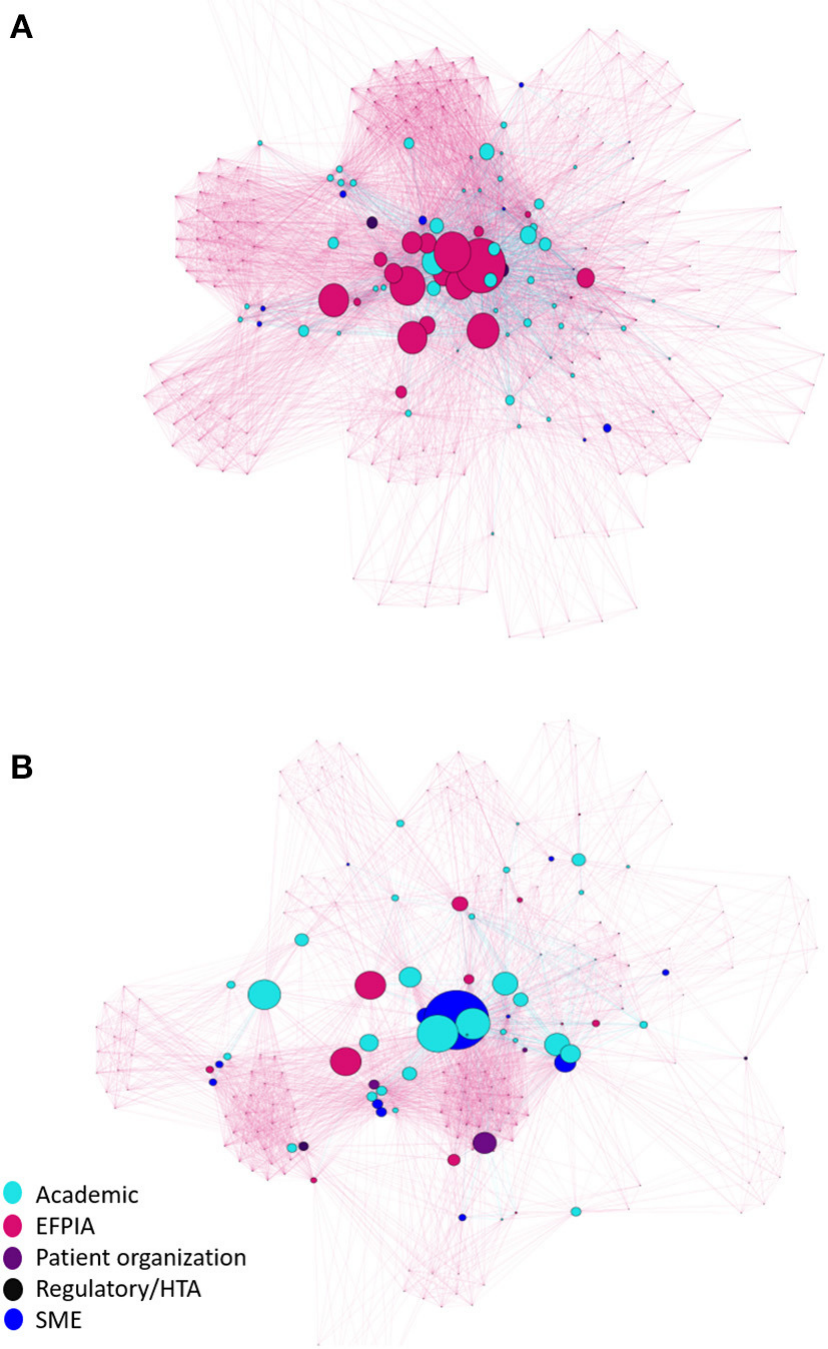
Network of unique partner organisations in the IMI NDD portfolio. **(A)** Network including EFPIA organisations. **(B)** Network excluding EFPIA organisations. Each organisation is represented by a single node, the size of which reflects how well-connected the organisation is with all the other organisations in the network (Betweenness Centrality). Lines connecting nodes are coloured according to the number of projects that connect individual organisations. Pink lines: participation in a single project. Blue lines: participation in 2 or more projects.

Figure 2A and Supplementary Figure 1 represents the network of all partner organizations across the IMI NDD portfolio. The results show the complexity of links across the network with a clear clustering of organizations at the center. The majority of organizations (*N* = 149) in the network are connected through participation in a single project, as indicated by the pink connections. There are a relatively small number of organizations that are the key nodes in the network, according to their betweenness centrality, as represented by the larger nodes in the visualization (Figure 2A and Table 3A). Of the top 20 organizations, 70% (*N* = 14) are EFPIA companies compared to just 5 academic institutions, in part due to the fact that there are many fewer EFPIA companies participating in IMI projects, compared to academic institutions, which make up 57% (*N* = 134) of the entire network. The majority (62%, *N* = 83) of academic organizations only participate in a single project. Janssen Pharmaceutica is the organization with the highest betweenness centrality in the network. This is partly the result of the large number of projects (*N* = 13) in which it participates and because it is also the biggest EFPIA contributor to the IMI NDD projects. None of the other organizations in the top 20 key nodes participate in more than nine projects.

**Table 3A:** Top 20 key nodes in the network analysis of the IMI NDD portfolio: Including EFPIA partners.

**Organization (Country)**	**Type**	**Projects (*N*)**	**Betweenness**	**Degree**
Janssen Pharmaceutica (BE)	EFPIA	13	1,437	197
UCB Biopharma (BE)	EFPIA	7	1,068	164
Pfizer (UK)	EFPIA	7	1,022	177
Novartis (BE)	EFPIA	9	928	140
AstraZeneca (UK)	EFPIA	5	870	108
Sanofi Aventis (FR)	EFPIA	7	847	149
Eli Lilly (UK)	EFPIA	8	807	132
Erasmus Medical Center (NL)	Academic	7	679	148
Biogen (UK)	EFPIA	5	617	117
Merck Sharp Dohme (BE)	EFPIA	4	568	126
F Hoffmann La Roche (SUI)	EFPIA	7	515	153
Takeda (UK)	EFPIA	6	513	130
H Lundbeck (DK)	EFPIA	7	502	109
Abbvie (FR)	EFPIA	5	465	101
Stichting VUMC (NL)	Academic	7	458	130
Kings College London (UK)	Academic	5	417	101
University of Cambridge (UK)	Academic	5	389	132
Academisch Ziekenhuis Leiden (NL)	Academic	4	375	100
Alzheimer Europe (LU)	Patient/carer organization	7	366	113
Amgen (SUI)	EFPIA	3	365	111

Figure 2B and Table 3B show the results of the network analysis for partner organizations in the IMI NDD portfolio, when EFPIA organizations are excluded from the analysis. As with the overall network, there is a relatively small number of organizations that are key nodes in network, according to their betweenness centrality. Of the top 20 organizations, 80% (*N* = 16) are academic institutions. Erasmus Medical Center is the top non-EFPIA organization in the network, with the highest betweenness, centrality and joint highest project participation (*N* = 7) with Alzheimer Europe and Stichting VUMC.

**Table 3B:** Top 20 key nodes in the network analysis of the IMI NDD portfolio: Excluding EFPIA partners.

**Organization (Country)**	**Type**	**Projects (*N*)**	**Betweenness**	**Degree**
Erasmus Medical Center (NL)	Academic	7	1,794	126
University of Cambridge (UK)	Academic	5	1,130	110
Stichting VUMC (NL)	Academic	7	946	109
Imperial College of Science, Technology and Medicine (UK)	Academic	3	891	75
Universitatsklinikum Erlangen (DE)	Academic	2	847	72
Academisch Ziekenhuis Leiden (NL)	Academic	4	843	82
University of Oxford (UK)	Academic	6	687	96
Alzheimer Europe (LU)	Patient/carer organization	7	683	92
Kings College London (UK)	Academic	5	639	81
Concentris Research Management (DE)	SME	3	598	75
Karolinska Institutet (SE)	Academic	6	579	86
VIB Center for Brain Disease Research (BE)	Academic	3	547	67
Parkinson's UK (UK)	Patient/carer organization	3	506	56
Charité Universitàtsmedizin Berlin (DE)	Academic	3	477	45
Stichting Katholieke Universiteit (NL)	Academic	5	428	59
University College London (UK)	Academic	4	393	71
University of Exeter (UK)	Academic	3	385	68
University of Sheffield (UK)	Academic	2	362	42
Mimetas (NL)	SME	3	360	38
Provincia Lombardo Veneta Ordineospedaliero di San Giovanni Di Dio Fatebenefratelli (IT)	Academic	2	330	66

When we assessed the degree centrality of organizations, we found that the minimum observed number of connections per organization across the whole network is 9, which means that every organization in the network is connected to at least nine other organizations. Janssen Pharmaceutica had the highest degree centrality (*N* = 197) which means that it is connected to 197 of 236 organizations in the IMI NDD portfolio (Table 3A). Excluding EFPIA organizations, the minimum observed number of connections per organization is 8. Erasmus Medical Center had the highest degree centrality (*N* = 126) which means that it is connected to 126 of 205 non-EFPIA organizations in the IMI NDD portfolio (Table 3B).

**Figure 3:**
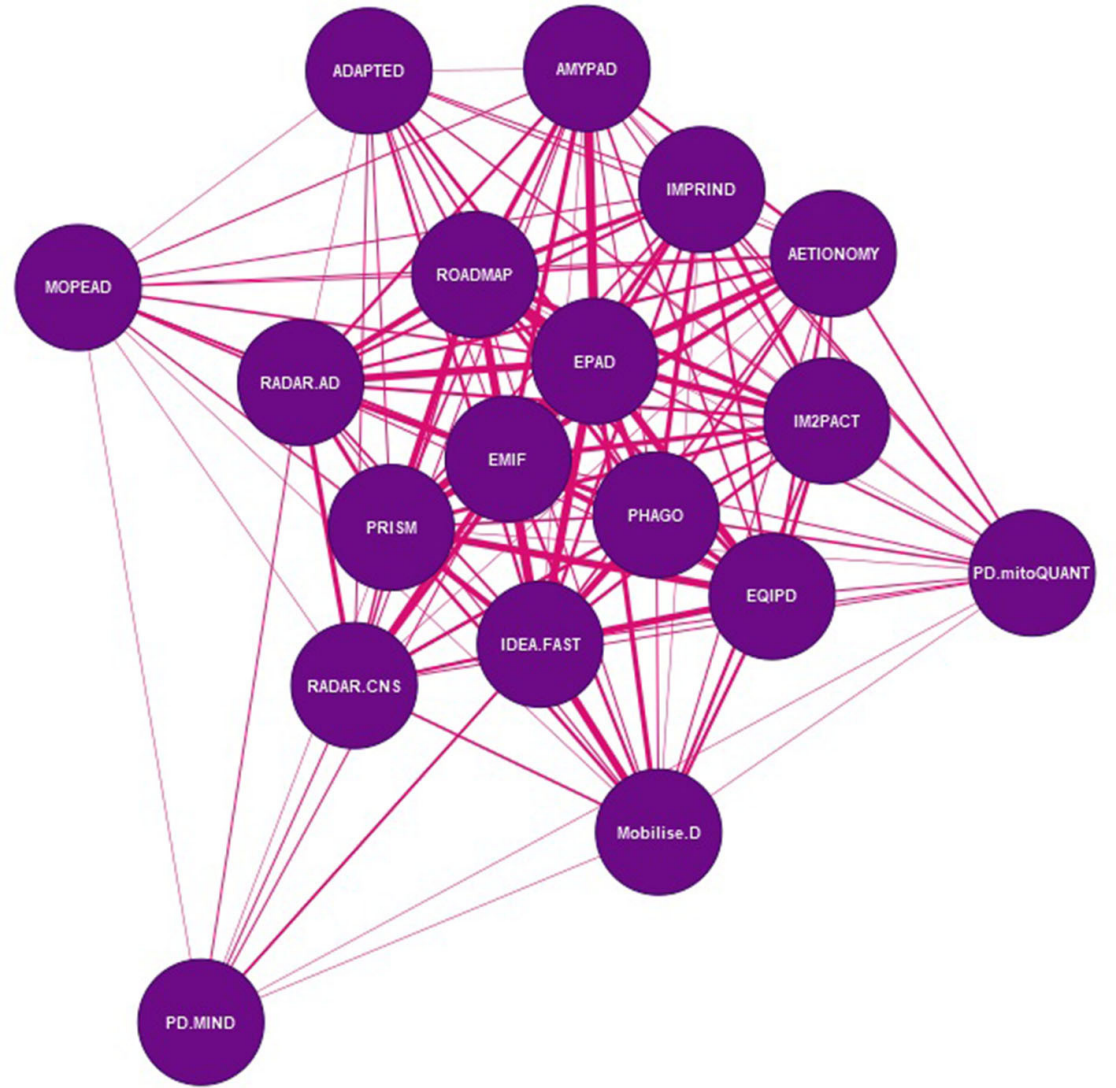
Network of shared organisations in IMI NDD projects. Each node in the network represents an IMI project. The lines between the nodes are weighted to show the number of organisations that participate in both projects – the wider the connector, the higher the number of shared organisations between projects.

Figure 3 shows the connections between projects across the whole network, where projects are connected by sharing at least one organization. The project with the lowest number of connections is PD-MIND which is connected to nine other IMI projects in the portfolio. All other projects are connected to at least 14 other IMI projects, with five projects (EMIF, IDEA-FAST, PHAGO, PRISM and RADAR-CNS) connected to all other projects in the portfolio through at least one partner organization (Table 4A). For each project we analyzed the proportion of project partner organizations that it shares with all other projects in the portfolio (Table 4A). Overall, we found that there are a number of projects that share multiple organizations with others, notably EPAD, EMIF and ROADMAP, which all address clinical research and/or real-world evidence. In contrast, other projects share far fewer organizations with the rest of the project portfolio, such as PD MIND and MOPEAD. When EFPIA organizations were excluded from the analysis, we found that the percentage of shared partner organizations between projects was reduced, confirming earlier results regarding EFPIA organizations being the core organizations across the network. However, there are some examples of projects that share a comparably high proportion of non-EFPIA organizations, including AMYPAD and EPAD, and ROADMAP and EMIF (Table 4B).

**Table 4A:** Percentage of project partners shared between IMI NDD projects (including EFPIA).

		**ADAPTED**	**AETIONOMY**	**AMYPAD**	**EMIF**	**EPAD**	**EQIPD**	**IDEA-FAST**	**IM2PACT**	**IMPRIND**	**Mobilise-D**	**MOPEAD**	**PD-MIND**	**PD-mitoQUANT**	**PHAGO**	**PRISM**	**RADAR-AD**	**RADAR-CNS**	**ROADMAP**	**Total number** **of projects** **connected to**
**Percentage of project partners shared with other IMI ND projects**	ADAPTED (*N* = 13 partners)		15%	15%	15%	31%	15%	31%	15%	15%	0%	15%	0%	8%	23%	15%	8%	15%	23%	15
	AETIONOMY (*N* = 16 partners)	13%		19%	31%	56%	25%	19%	19%	6%	13%	13%	0%	13%	19%	19%	25%	6%	19%	16
	AMYPAD (*N* = 15 partners)	13%	20%		40%	80%	20%	7%	13%	7%	0%	20%	0%	13%	13%	20%	27%	13%	40%	15
	EMIF (*N* = 60 partners)	3%	8%	10%		27%	12%	18%	10%	8%	7%	5%	3%	3%	10%	13%	10%	10%	17%	17
	EPAD (*N* = 39 partners)	10%	23%	31%	41%		23%	26%	21%	15%	15%	10%	0%	8%	21%	26%	23%	15%	36%	16
	EQIPD (*N* = 29 partners)	7%	13%	10%	23%	30%		27%	17%	13%	17%	0%	0%	10%	20%	30%	7%	10%	17%	15
	IDEA-FAST (*N* = 51 partners)	8%	6%	2%	22%	20%	16%		8%	8%	18%	4%	6%	4%	16%	14%	6%	6%	14%	17
	IM2PACT (*N* = 27 partners)	7%	11%	7%	22%	30%	19%	15%		22%	15%	0%	0%	7%	11%	19%	15%	11%	26%	15
	IMPRIND (*N* = 18 partners)	11%	6%	6%	28%	33%	22%	22%	33%		6%	11%	0%	17%	33%	17%	22%	11%	33%	16
	Mobilise-D (*N* = 36 partners)	0%	6%	0%	11%	17%	14%	25%	11%	3%		3%	3%	3%	6%	11%	6%	8%	6%	15
	MOPEAD (*N* = 15 partners)	13%	13%	20%	20%	27%	0%	13%	0%	13%	7%		7%	0%	13%	7%	20%	7%	13%	14
	PD-MIND (*N* = 10 partners)	0%	0%	0%	20%	0%	0%	30%	0%	0%	10%	10%		10%	20%	10%	20%	20%	0%	9
	PD-mitoQUANT (*N* = 14 partners)	7%	14%	14%	14%	21%	21%	14%	14%	21%	7%	0%	7%		21%	7%	0%	14%	7%	15
	PHAGO (*N* = 20 partners)	15%	15%	10%	30%	40%	30%	40%	15%	30%	10%	10%	10%	15%		15%	20%	20%	25%	17
	PRISM (*N* = 23 partners)	9%	13%	13%	35%	43%	39%	30%	22%	13%	17%	4%	4%	4%	13%		22%	13%	35%	17
	RADAR-AD (*N* = 16 partners)	6%	25%	25%	38%	56%	13%	19%	25%	25%	13%	19%	13%	0%	25%	31%		38%	44%	16
	RADAR-CNS (*N* = 25 partners)	8%	4%	8%	24%	24%	12%	12%	12%	8%	12%	4%	8%	8%	16%	12%	24%		12%	17
	ROADMAP (*N* = 26 partners)	12%	12%	23%	38%	54%	19%	27%	27%	23%	8%	8%	0%	4%	19%	31%	27%	12%		16

**Key:**

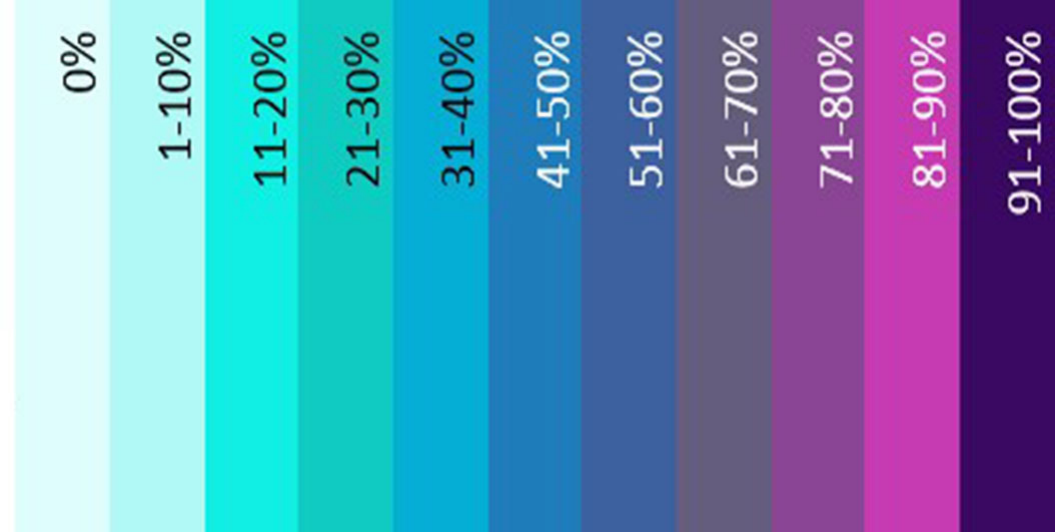

**Table 4B:** Percentage of project partners shared between IMI NDD projects (excluding EFPIA).

		**ADAPTED**	**AETIONOMY**	**AMYPAD**	**EMIF**	**EPAD**	**EQIPD**	**IDEA-FAST**	**IM2PACT**	**IMPRIND**	**Mobilise-D**	**MOPEAD**	**PD-MIND**	**PD-mitoQUANT**	**PHAGO**	**PRISM**	**RADAR-AD**	**RADAR-CNS**	**ROADMAP**	**Total number** **of projects** **connected-to**
**Percentage of project partners shared with other IMI ND projects**	ADAPTED (*N* = 10 partners)		20%	10%	10%	20%	0%	10%	10%	0%	0%	20%	0%	10%	10%	10%	0%	0%	10%	10
	AETIONOMY (*N* = 12 partners)	17%		25%	25%	42%	0%	8%	8%	0%	0%	17%	0%	8%	17%	8%	25%	0%	17%	12
	AMYPAD (*N* = 12 partners)	8%	25%		42%	92%	17%	0%	8%	0%	0%	25%	0%	17%	8%	17%	25%	8%	33%	12
	EMIF (*N* = 50 partners)	2%	6%	10%		18%	2%	14%	6%	6%	2%	6%	4%	2%	8%	8%	10%	6%	16%	17
	EPAD (*N* = 24 partners)	8%	21%	46%	38%		8%	8%	13%	8%	0%	13%	0%	4%	8%	13%	21%	4%	25%	15
	EQIPD (*N* = 18 partners)	0%	0%	11%	6%	11%		6%	6%	0%	6%	0%	0%	6%	6%	22%	0%	6%	11%	11
	IDEA-FAST (*N* = 40 partners)	3%	3%	0%	18%	5%	3%		3%	3%	13%	0%	5%	3%	3%	5%	0%	0%	5%	13
	IM2PACT (*N* = 20 partners)	5%	5%	5%	15%	15%	5%	5%		15%	5%	0%	0%	5%	0%	10%	10%	5%	20%	14
	IMPRIND (*N* = 11 partners)	0%	0%	0%	27%	18%	0%	9%	27%		0%	9%	0%	18%	18%	0%	9%	0%	18%	9
	Mobilise-D (*N* = 24 partners)	0%	0%	0%	4%	0%	4%	21%	4%	0%		0%	0%	0%	0%	4%	0%	8%	0%	6
	MOPEAD (*N* = 13 partners)	15%	15%	23%	23%	23%	0%	0%	0%	8%	0%		0%	0%	0%	0%	15%	8%	8%	9
	PD-MIND (*N* = 9 partners)	0%	0%	0%	22%	0%	0%	22%	0%	0%	0%	0%		11%	11%	11%	22%	22%	0%	7
	PD-mitoQUANT (*N* = 14 partners)	9%	9%	18%	9%	9%	9%	9%	9%	18%	0%	0%	9%		18%	9%	0%	0%	0%	12
	PHAGO (*N* = 11 partners)	9%	18%	9%	36%	18%	9%	9%	0%	18%	0%	0%	9%	18%		0%	18%	18%	9%	13
	PRISM (*N* = 16 partners)	6%	6%	13%	25%	19%	25%	13%	13%	0%	6%	0%	6%	6%	0%		6%	13%	19%	14
	RADAR-AD (*N* = 12 partners)	0%	25%	25%	42%	42%	0%	0%	17%	8%	0%	17%	17%	0%	17%	8%		42%	25%	12
	RADAR-CNS (*N* = 20 partners)	0%	0%	5%	15%	5%	5%	0%	5%	0%	10%	5%	10%	0%	10%	10%	25%		0%	11
	ROADMAP (*N* = 17 partners)	6%	12%	24%	47%	35%	12%	12%	24%	12%	0%	6%	0%	0%	6%	18%	18%	0%		13

**Key:**

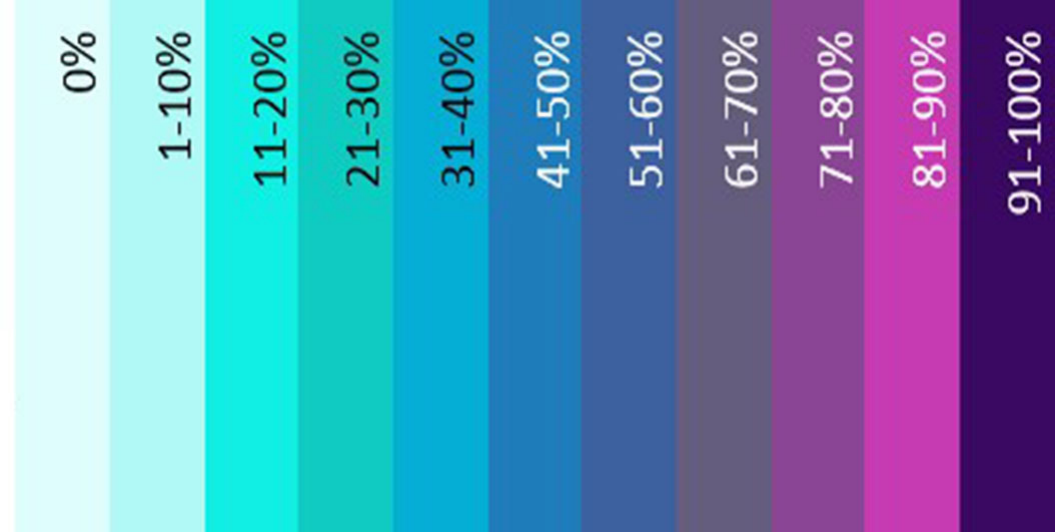

### Collaborations, challenges and opportunities

Overall, a response rate of 100% (16/16) was received for the survey of past collaborations. The results identified nine past collaboration attempts of which six were materialized (totally or partially) and three were unsuccessful. Overall, the projects reported that the main obstacle for collaboration was the need for collaboration agreements between projects or other legal requirements which led to lengthy delays in the sharing of data, often meaning that the data was shared too late for the collaborating projects' requirements.

**Figure 4:**
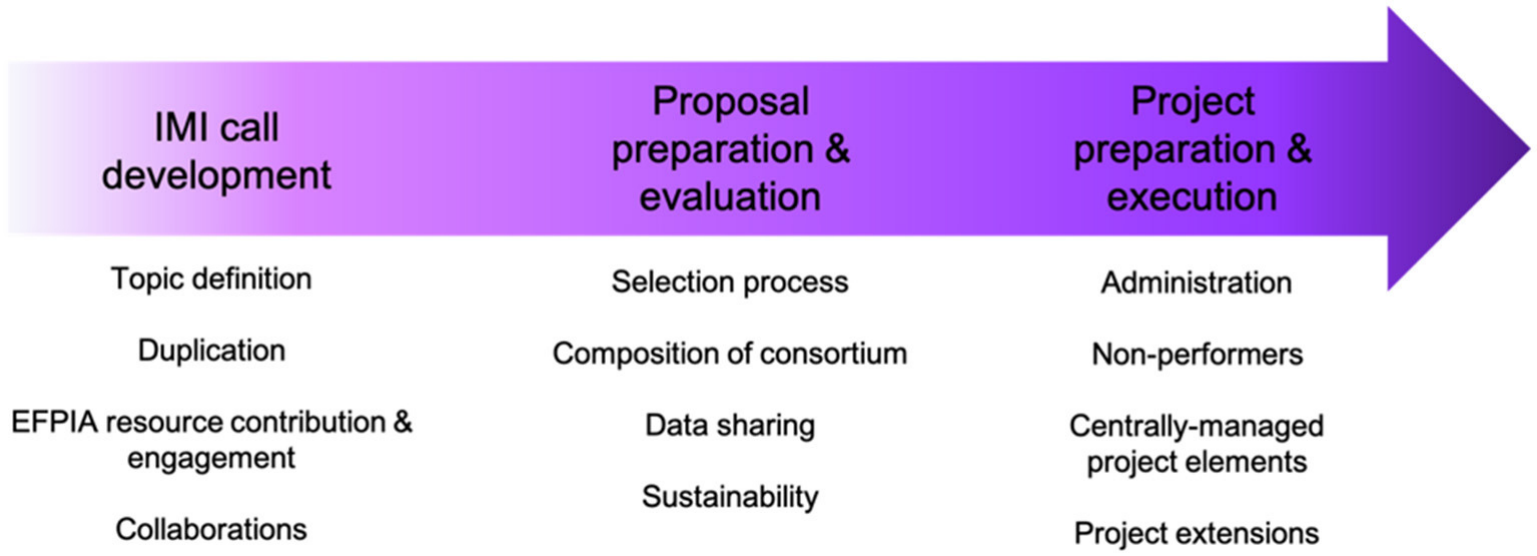
Challenges and opportunities for improvement across the IMI project lifespan.

Together, the responses from the survey and the multiple interviews held with project leaders across the whole portfolio identified 11 main themes (Figure 4) in relation to the challenges and opportunities for improvement across the three stages of an IMI project.

### Before the call launch and topic development

#### Topic definition

The legal framework with regards to the Intellectual Property (IP) of IMI projects and financial rules have not always been found to be most suitable for all topics spanning the target identification and drug development pipeline. Pure fundamental research projects in the precompetitive space seem more feasible to execute compared to projects in the gray zone between precompetitive and competitive space. For example, projects aiming to develop platforms for studies or clinical trials of drugs that rely on different industrial IP holders providing compounds to run studies under a single academic sponsor. In such cases, the operational set-up of the site network, study and trial platform (both on legal and financial grounds) within the IMI1 legal and financial framework can prove to be quite challenging and time-consuming, resulting in delays that could affect e.g., conformity to meet the timelines from IP holders.

#### Duplication

As indicated above, the 18 projects of the IMI NDD portfolio have developed a wide range of assets and outputs that could be of value to the NDD research community and other stakeholders. There is a need to improve the sharing of information about this wealth of assets, in order to de-risk investment being made in duplicative efforts, as well as to inform projects about the key lessons learned from the development of these assets. For example, for some IMI NDD projects, it would have been more useful to make use of existing cohorts, such as the EPAD longitudinal cohort, which formed the backbone of the AMYPAD clinical studies, instead of creating new ones.

#### EFPIA resource contribution and engagement

The intended resource contribution of some EFPIA partners in IMI projects does not always translate to active engagement, as priorities and personnel within organizations may change during the project duration. This can have an impact on the involvement of EFPIA or Associated partners and their actual resource contribution.

#### Collaborations – organically grown vs. imposed

Interdependencies with other calls/projects are often written in topic texts, as well as in short proposals. It is not always clear whether these collaborations are a critical dependency, or something that is just simply desirable. In the case of those that are critically dependent, separate Grant Agreements with distinct timelines, budget and objectives are typically difficult to reconcile. The intention to collaborate through ‘letters of support’ are often not realized due to a lack of assessment of feasibility and the resources needed to implement such a collaboration.

### Call launch, 2-stage submission and evaluation of project proposals

#### Selection process

The selection process for IMI projects involved two distinct stages. The first stage, during which an academic consortium was formed, with each partner assuming defined roles based on well-specified budgets, culminated in the selection of a single successful consortium, based on proposal ranking by external reviewers. EFPIA partners joined the proposal at the second stage, with consortia adapting, extending and optimizing the initial first-stage proposal to include their contributions. As a result of this two-stage process, collaboration between EPFIA and Academic partners is not always optimal and could be improved. In particular, as EFPIA partners are not involved in the selection of the winning application, they may end up in a collaboration with an academic partner (the selected applicant consortium) that is not always an optimal complement.

#### Stakeholders

Having large numbers of project partners increases the risk of a project becoming unwieldy, with large internal overheads (e.g. administrative) and a greater risk of absent or silent partners. This may impact overall project efficiencies and getting true value for money.

#### Sustainability

The ultimate impact of most IMI projects depends on its capacity to guarantee uptake of its results and to fully leverage the value of its assets beyond the funding period of the project. However, most consortia struggle to develop credible plans for sustainability. Sustainability activities are challenging for several reasons, including: Consortia not being legal entities themselves; sustainability activities after the project period falling outside the Grant Agreement and therefore requiring a *de novo* commitment from interested parties; a disconnect within institutions between the principal investigators and decision-makers in terms of long-term commitment; and a lack of knowledge and experience within consortia about business planning, assessment and set up, leading to an inappropriate analysis of the value of assets and of the ways in which these could be sustained.

There is a trend to alleviate these challenges through the consideration of sustainability aspects at the beginning of projects, or even before they start. However, this does not necessarily increase buy-in or uptake by potential funders or customers, particularly because of the inherent risks of collaborative, distributed research efforts prevail until results are solid enough to gauge their exploitation potential, which typically occurs during the second half of any project.

### Project preparation and execution

#### Administration

Administrative requirements within the IMI framework are generally considered as being quite cumbersome. Legal documents/procedures (e.g., Grant and Project/Consortium Agreements) are time-consuming to complete and can lead to the excessive use of templates and default conditions that are not adapted to the project's reality. During project execution, in order for two projects to share results, assets, confidential information and/or other solutions, all beneficiaries may need to approve and sign a dedicated collaboration agreement. This can be a very time-consuming process causing major delays and sometimes undermining timely collaboration.

#### Non-performers

Our surveys and interviews found that some project leaders felt that having an easier way out for non-performing partners would be beneficial in an IMI project. Coordinators or Leads do not always have enough leverage to remove non-performing partners, and are faced with challenges in reallocating budgets/tasks and formalizing the required amendments to grant agreements.

#### Data sharing

Data sharing between both public and private partners within the context of a PPP does not always materialize in practice. For example, partners are not always fairly acknowledged when sharing data with others. This acknowledgment should reflect their efforts in collecting the data, as well as the efforts required to manage the burdensome administrative and legal processes that underpin secure, ethical data sharing.

#### Centrally managed project elements

Some project elements could be managed centrally (e.g., by IMI) through the provision of key tools, such as communications plans, technical solutions (e.g., website platforms) and project management tools. This would allow for a more efficient use of resources and would centralize project information and data, without the risk of information being lost when an individual project ends.

#### Project extensions

Whilst requests for additional time or resources at the end of the initial IMI project are common and enable consortia extra time, and in some cases extra resources to finalize the development of an asset or to make the asset sustainable, the possibility of, and process for allowing extensions would benefit from being more transparent.

## Discussion

The IMI NDD portfolio represents a complex landscape of research projects implemented through public-private partnerships across multiple NDD areas, with a strong focus on Alzheimer's disease and a secondary focus on Parkinson's disease. The breadth of research being undertaken ranges from preclinical studies in cells and animals, translational work with samples and data from patients and participants, clinical studies including longitudinal cohort studies and clinical trials, and the development and testing of digital biomarkers.

Our findings show that the IMI NDD portfolio has contributed to the development of tools, standards and approaches to address the high unmet medical need for effective disease-modifying as well as symptomatic interventions in NDDs in general, and Alzheimer's disease in particular. For example, IMI projects such as EMIF and EPAD have developed platforms and infrastructures to speed up clinical development, also generating cohort datasets which have been widely used by researchers to advance the development of novel, non-invasive biomarkers for the diagnosis and monitoring of Alzheimer's disease from its very earliest stages (6, 7). The EQIPD Quality System, which includes a series of tools, guidance and requirements to support preclinical researchers ensure their work is robust and reliable, is being incorporated into the global Partnership for Assessment and Accreditation of Scientific Practice (PAASP) network (https://paasp.net). Together, the RADAR-CNS and RADAR-AD projects have developed and refined the RADAR-Base system (https//radar-base.org), an open-source platform for remote assessment using wearables and mobile applications, which is now also being used by external groups for studies on remote monitoring of lung diseases (8). While research and innovation efforts such as these have opened new commercial possibilities based on new services and products, IMI efforts have been especially beneficial in terms of scientific progress and publishable results. It is also important to note that IMI NDD projects also provide intangible benefits, such as support for early career researcher training and development, as well as greater interaction and coordination across industry, academia and other sectors. Our analyses clearly show that the research, industry and societal sectors involved in IMI have benefited from the cooperation and knowledge sharing that take place in IMI projects. This has yielded a situation where collaboration across competing companies and researchers is seen as a natural thing and not as an exception.

Previous studies have highlighted challenges in assessing the performance and impact of PPPs in the life sciences (9). As shown by our analyses of the IMI NDD portfolio, PPP projects often have timelines of 4–6 years, aiming to impact lengthy drug development pipelines that can take decades to reach maturity. Moreover, the value of PPPs extends to parameters that are hard to measure quantitively, such as knowledge transfer, educational aspects and collaboration. Nevertheless, the number of PPPs launched per year has grown over time (from 8 in 2001–2003 to 54 in 2011–2013) (9) and analyses of research publications from IMI projects show that almost 60% of these are published in journals with a high impact factor (IF) (10–12). Editorials have highlighted how IMI projects are developing new regulatory tools and pathways to facilitate interactions with regulatory bodies, helping to identify and address obstacles to regulatory approval (13). These and other publications illustrate the value of the IMI model of research and development as a driver of innovation to address unmet clinical needs. Our findings provide further evidence to support this, highlighting the multiple benefits and positive impacts arising from IMI projects on NDD.

Across the IMI NDD portfolio there is a complex network of partner organizations, each with the potential to enable the exchange of new knowledge and tools within and between projects. We found that there is a relatively small number of organizations that are central to the IMI NDD portfolio, both in terms of the number of connections they have to all other organizations in the network and the connections they form between organizations. The majority of these key organizations participate in the largest projects in the portfolio and form the key links between different IMI projects. Unsurprisingly, EFPIA companies make up the largest percentage of these organizations, reflecting the intrinsic role that EFPIA have in the IMI model and the relatively small pool of EFPIA organizations from which participation can be drawn. Whilst academic organizations represent the largest group of stakeholders in the portfolio, they are also the most diverse: there are relatively few academic institutions that are involved in multiple projects, despite the portfolio representing the same overall field of research.

The key organizations in the network, particularly EFPIA companies, may have the greatest opportunities to create synergies and ensure the dissemination of knowledge, tools, methods and experience across the portfolio compared to other organizations whose involvement in multiple IMI projects is more sporadic. However, these organizations are frequently global entities with multiple departments and people involved across different projects thus making dissemination across the portfolio less likely.

On a project level, there is some clustering of groups of organizations who collaborate more frequently across multiple projects. These are generally projects that are focused on the study of Alzheimer's disease and are clinically driven, such as ROADMAP and EPAD, whilst other projects in the portfolio, such as PD MIND and MOPEAD, share comparably fewer organizations with other projects. For projects such as these, the lower number of connections to the rest of the network could potentially limit their ability to disseminate and leverage the new knowledge that is being generated within these projects and thus limit their potential impact.

NEURONET has attempted to address many of these challenges through a systems leadership type approach, promoting integration, knowledge transfer and cohesion across the portfolio, suggesting and supporting new collaborations, and facilitating the dissemination of project results both across and beyond the portfolio.

Along with these challenges, our analyses have identified a number of key lessons learnt from past collaborations. Firstly, to facilitate the operational setup of IMI projects, the existing IMI IP and financial guidelines could be adapted. As the IP clauses in the IMI2 model Grant Agreement leave some room to maneuver (e.g., 23a.1, “Beneficiaries…must take measures to implement the principles set out in points 1 and 2 of the Code of Practice”), the development of specific, but adaptable template documents for IMI projects in precompetitive and competitive spaces could be extremely valuable, whilst leaving enough flexibility to projects to be creative in how financial structures/flows serve project progress best. Any risks that this flexibility create could be managed by e.g., clearly set milestones or go/no-go points defined in advance.

To de-risk duplicative efforts in new IMI NDD projects, communication between IMI NDD projects could be improved from even the application stage, and greater connections could be created between projects and the IMI Strategic Governing Group (SGG), that was responsible for instigating new call topics. Concerning IMI projects with less innovative technologies, a balanced approach could be to place huge bets on high-risk, disruptive or discontinuous innovation whilst also funding sustainable and continuous innovation. For example, this could be done by building on or maintaining valuable portfolio assets that have already been developed. For high-value portfolio assets, IMI could play an important role in helping projects bridge the gap toward sustainability, for example through conditional funding mechanisms that allow for extended grants renewable under the condition of tangible results being obtained.

To ensure that the commitment of some EFPIA partners in IMI projects is meaningful, more strict rules should be defined (e.g., by ensuring more specific/balanced task allocation, or by adapting the IMI mid-term review process to detect and remedy “absent” partners). These rules could be implemented *via* Memoranda of Understanding (MoUs) between the steering committees of IMI projects and/or project partners (replacing traditional “letters of support”) at the design stage, coupled with more precise collaboration agreements before signature of Grant Agreements. It may also be advisable to encourage more detailed contingency planning, extending to the identification of alternative datasets, and sources of material in case collaboration cannot be implemented, to avoid extreme dependency. This should be done in a way that doesn't hinder any potential partnerships, and that doesn't impose an unmanageable administrative burden at the application stage and at the delicate initial stages of implementation. It may also need identification of mutual incentives for collaboration *ex ante* to avoid excessive name-dropping in call texts that may be interpreted as pre-requisite. An additional recommendation might therefore be to be clearer about why other projects are mentioned in call texts and what collaboration is exactly expected of applicant consortia in that respect.

The quantity, value and impact of assets described above underlines the importance of ensuring timely and effective sustainability planning for IMI project outputs such as these. A first option could be to formalize the requirement for a “sustainability fund” to be set aside by a consortium for each new IMI project. Another possibility might be to create a central “sustainability fund” at IMI. The central sustainability fund at IMI could be dedicated to the asset maintenance of IMI projects, enabling a transition from project to self-sustainability status. Similarly, central structures (databases) for data assets could be set up, including mechanisms for access to federated resources, data discovery, etc. that act as reference point for current and future projects. Ideally, the legal and practical terms for sharing of resources, data and know-how should be formalized from the start of a project (e.g., endorsing the Data Citation Principles, ensuring that data declaration of interests (DOIs) are appropriately used, providing specific guidance for biobanking and data storage, etc).

Our findings may prove useful for the forthcoming Innovative Health Initiative and other EU funding entities in shaping the next calls and framework programmes. Furthermore, NEURONET offers a unique role in providing an integrated view of IMI funded NDD research, facilitating synergies and collaboration, disseminating results and ensuring the sustainability of tangible assets beyond the duration of a project. In this role, NEURONET could bridge the gap between IMI and IHI, and provide a model of portfolio management that could be reproduced in other research areas.

In conclusion, our analysis reveals a complex landscape of IMI NDD projects covering the breadth of research and disease stages, with over 200 partner organizations from 24 different countries. Despite this complexity, our analysis identified multiple connections between organizations and projects. Whilst our analysis has not sought to understand whether these connections have led to the dissemination of information between projects, it does highlight the potential role of key organizations to facilitate the exchange of new knowledge and promote the uptake of tools and assets developed by individual IMI projects. Our findings also underline the value of systems leadership approaches in identifying and addressing complex challenges for research on neurodegenerative diseases. Since NEURONET was established in 2019, it has focused on boosting the visibility and impact of projects and identifying and supporting new cross-project synergies and collaborations. By analyzing the structure of the IMI NDD portfolio and previous collaboration attempts, NEURONET has been able to identify links and potential new synergies and collaborations, as well as providing recommendations that could help increase the efficiency and impact of future public-private partnerships on neurodegeneration.

## Data availability statement

The raw data supporting the conclusions of this article will be made available by the authors, without undue reservation.

## Author contributions

DO'R, NC-P, and AB performed data collection, content analysis, and drafted the manuscript. LK and DO'R performed network analyses and developed manuscript figures. LS and CD conducted interviews. JG coordinated the Alzheimer Europe conference at which project presentations were delivered. LP, JG, DD, LS, and CD coordinated the project. All authors provided critical comments on the manuscript draft and approved the final version of the manuscript.

## Funding

This work was supported by funding from the Innovative Medicines Initiative 2 Joint Undertaking (JU) under grant agreement number 821513 (NEURONET). The IMI JU receives support from the European Union's Horizon 2020 research and innovation programme and EFPIA, and the Parkinson's Disease Society of the UK LBG.

## Conflict of interest

Authors NC-P, LK, and CD were employed by SYNAPSE Research Management Partners. Author LP was employed by Sanofi. Author LS was employed by Janssen Pharmaceutica NV. The remaining authors declare that the research was conducted in the absence of any commercial or financial relationships that could be construed as a potential conflict of interest.

## Publisher's note

All claims expressed in this article are solely those of the authors and do not necessarily represent those of their affiliated organizations, or those of the publisher, the editors and the reviewers. Any product that may be evaluated in this article, or claim that may be made by its manufacturer, is not guaranteed or endorsed by the publisher.

## Footnotes

^1^AETIONOMY, AMYPAD, EMIF, EPAD, EQIPD, IM2PACT, MOPEAD, PHAGO, PRISM, RADAR-AD, ROADMAP.
