# Supplementary figures and images for: The Innovative Medicines Initiative neurodegeneration portfolio: From individual projects to collaborative networks
Source: Front Neurol. 2022 Nov 2;13:994301. doi: 10.3389/fneur.2022.994301 (PMC9666729; doi:10.3389/fneur.2022.994301)

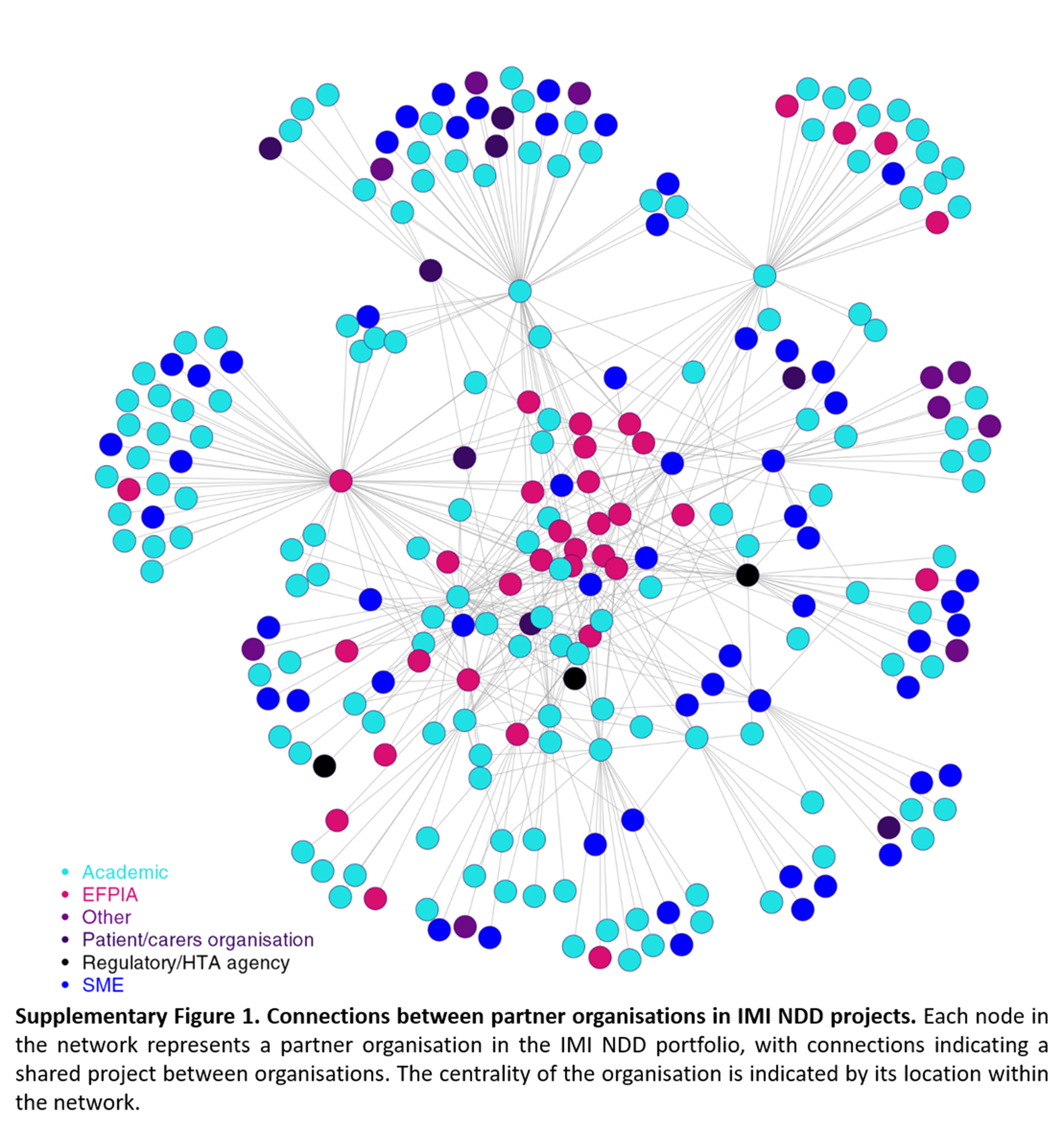

Supplement: Supplementary file 1 [file Image_1.TIF]

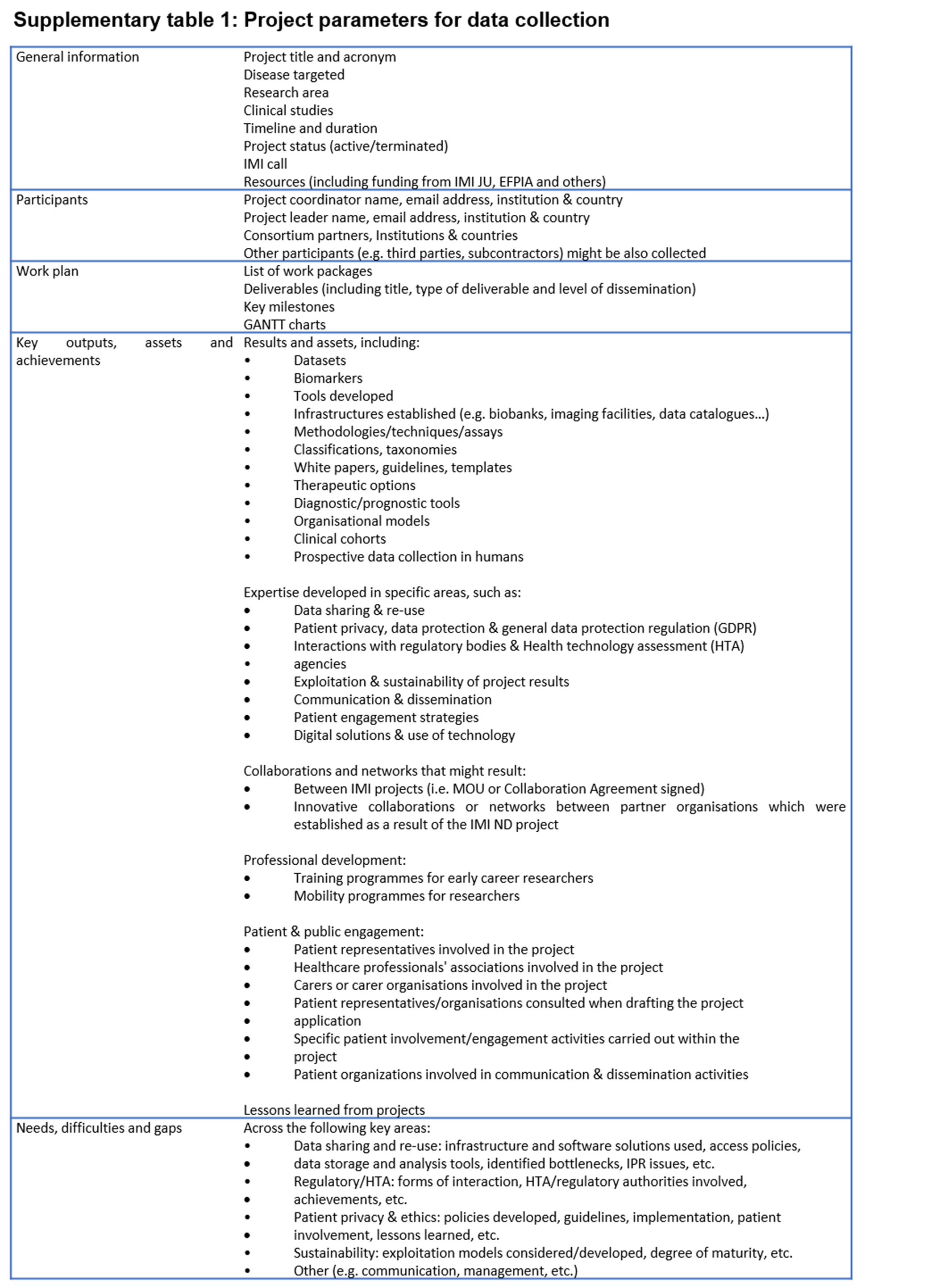

Supplement: Supplementary file 2 [file Image_2.TIF]
